# Compound Presentation

**Published:** 1871

**Authors:** 


*Compound Presentation.*

In presentation of the head with the hand or foot, Prof. R. Coleman recommends, in the *Virginia Clinical Record*, the following treatment:

The accoucheur should never be satisfied with ascertaining that the head presents, but should sweep with the finger the whole field of the os uteri, in order that he may positively determine that the head alone presents, and not the head with the hand, foot, or funis.

Timely information when these complications exist will lead to their speedy and easy correction, and thus save much subsequent trouble to the accoucheur, suffering to the mother, and danger to the child. Bearing in mind the possible occurrence of such complications, the doctor should be careful not to infer that there necessarily exists a presentation of the trunk, because he finds a hand in the vagina; nor a presentation of the pelvis, because he discovers a foot, until he has ascertained that the head is not also presenting. Not much can be done toward a correction of the complication under consideration, until towards the close of the first, or the commencement of the second stage of labor. Before the head has passed the circle of the os uteri, the indication is, of course, by judiciously applied pressure, during *the absence of pain,* to replace the displaced limb, pushing it above the parietal protuberance, and holding it there until a pain comes on, and forces the head to monopolize the circle of the os uteri, thus effectually preventing the re-descent of the hand or foot.

This object is secured additionally, by rupturing at this time, if not previously ruptured, the bag of waters. If the head has passed the os uteri, then no effort should be made to replace the prolapsed part until the head approaches the inferior strait, when well applied pressure (that is, in the direction opposite to that of the displacing force, so that the part can be returned by reversing the order of its displacement,) will cause its ascent in the direction of the face, above the head, in the now distended and elongated vaginal canal. We cannot interfere after the head has passed the os uteri, but is not yet about to engage in the inferior strait, for the simple reason that the prolapsed llmb cannot now be returned into the uterus, and any effort to accomplish this might lacerate the uterus; nor is there room enough as yet in the vagina for its return above the head. All that the practitioner can do under these circumstances is to carry the limb towards the temporal or malar region, so that it will engage with the shortest diameters of the fœtal head. When the head is about to engage in the inferior strait, and the manœuvre for the return of the limb, above indicated, cannot be accomplished, then if the head advances with each recurring pain, as sometimes happens when the head is small and the pelvis capacious, the case may be left to nature. If, however, there be no advance, and the child he still alive, the forceps should be applied, taking care that the blade be cautiously carried between the head and the prolapsed leg or arm, and the head brought down with its short diameters in apposition with the long diameters of the pelvis.

Should this procedure fail, or the child be dead, then the head should be perforated, and with its diameters thus reduced, it can be forced through the pelvis by the uterine contractions; or, if these prove inefficient, it can be drawn through either by the tractor, crochet, or blank hook, but better still, by the forceps applied as previously directed.

The foregoing directions are equally applicable, whether the head be engaged with the arm or leg. There is, however, one material difference in the management of these respective complications. If the head has not passed the circle of the os uteri, and the leg or foot be engaged along with it, should efforts to return the prolapsed part be unsuccessful, then the accoucheur, by traction on the leg, and pushing up the head at the same time, can convert this case into a footling.—*Med. and Surg. Reporter.*

